# Combined electrochemical and spectroscopic investigations of carbonate-mediated water oxidation to peroxide

**DOI:** 10.1016/j.isci.2024.109482

**Published:** 2024-03-11

**Authors:** Hossein Bemana, Nikolay Kornienko

**Affiliations:** 1 Department of Chemistry, Université de Montréal, 1375 Avenue Thérèse-Lavoie-Roux, Montréal, QC H2V 0B3, Canada; 2 Institute of Inorganic Chemistry, University of Bonn, Gerhard-Domagk-Str. 1, 53121 Bonn, Germany

**Keywords:** Catalysis, Electrochemistry, Spectroelectrochemistry

## Abstract

The development of electrosynthetic technologies for H_2_O_2_ production is appealing from a sustainability perspective. The use of carbonate species as mediators in water oxidation to peroxide has emerged as a viable route to do so but still many questions remain about the mechanism that must be addressed. To this end, this work combines electrochemical and spectroscopic methods to investigate reaction pathways and factors influencing the efficiency of this reaction. Our results indicate that CO_3_^2−^ is the key species that undergoes electrochemical oxidation, prior to reacting with water away from the catalyst. Through spectroelectrochemical experiments, we noted that CO_3_^2−^ depletion is a factor that limits the selectivity of the process. In turn, we showed how the application of pulsed electrolysis can augment this, with an initial set of optimized parameters increasing the selectivity from 20% to 27%. In all, this work helps pave the way for future development of practical H_2_O_2_ electrosynthetic systems.

## Highlights

• Mechanism of two-electron water oxidation to peroxide investigated
• Carbonate ions play a key role as a mediator in the reaction mechanism
• Controlling near-surface carbonate concentration was key to enhancing performance

## Graphical abstract

**Figure:**
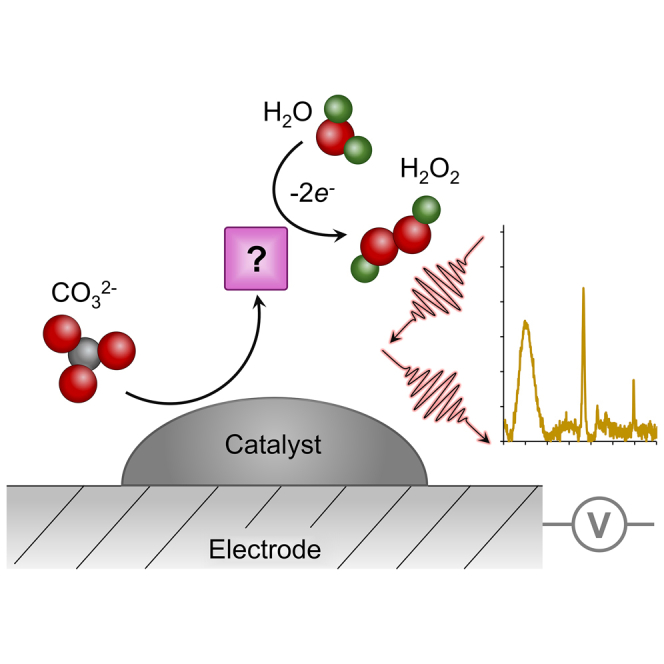

## Introduction

Hydrogen peroxide is a widely used oxidant, which has the highest oxygen content by weight among the peroxides and is effective in the whole pH range. Employing H_2_O_2_ in a reaction generally results in oxygen and water as the main byproducts, rendering it a potentially green oxidant.^1^ H_2_O_2_ is conventionally produced by the anthraquinone process which possesses limitations of safety and a substantial carbon footprint.^2^ In particular, this process uses H_2_ produced from the steam reforming of methane, a particularly CO_2_-emissive process.^1^

An alternate route to H_2_O_2_ production is through electrocatalysis.^3^ The appeal to electrocatalytic H_2_O_2_ production is that the process can ideally be powered by renewable electricity at a variety of scales. Furthermore, electrochemical systems are inherently modular as they do not adhere to the same scaling relations as thermochemical processes and thus, electrocatalytic peroxide production can be carried out in a distributed fashion, producing it at the point of use. This is greatly beneficial for applications like water purification in remote communities.^4^ In addition, H_2_O_2_ production at one electrode can be combined with other reactions at the counter electrode to provide dual product streams of value-added chemicals in a modular fashion.^5^^,^^6^^,^^7^^,^^8^

**Figure 1:**
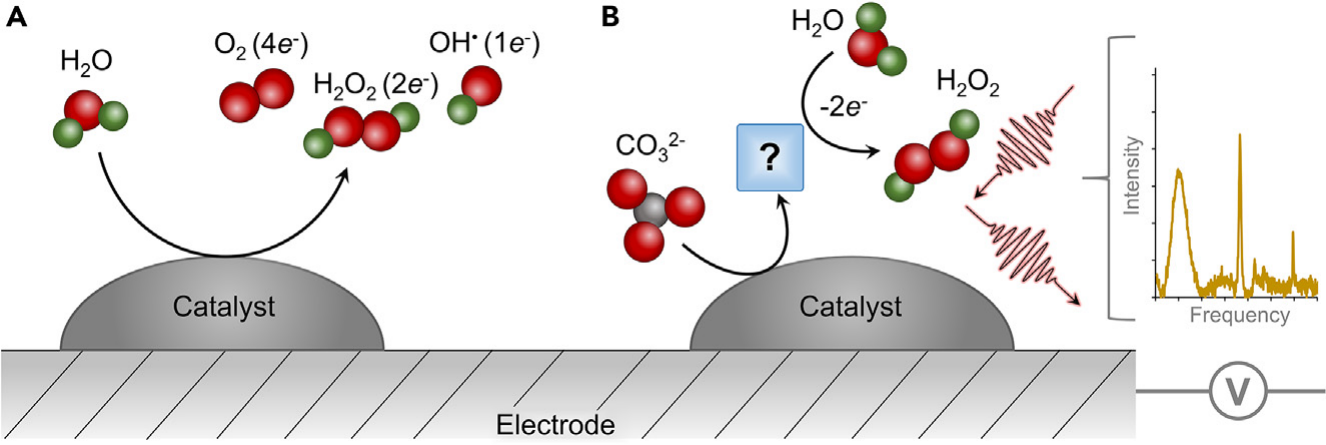
General scheme Direct water oxidation reaction pathways (A) and our approach to help elucidate carbonate-mediated peroxide formation (B).

To this end, there are two main catalytic routes: anodic 2-electron water oxidation reaction^9^ (2*e*^−^WOR, Equation 1) or cathodic oxygen reduction reaction^10^ (2*e*^−^ORR, Equation 2), which may be performed within a single electrolyzer in parallel. A significant bottleneck lies within the anodic side, predominantly arising from competition with the 4*e*^*-*^ WOR (Equation 3) (Figure 1A).

(Equation 1) $$2H_{2}O\;↔\;H_{2}O_{2}+2H^{+}+2e^{-}\;E^{0}=1.76\;V\;\text{vs}.\;\text{RHE}$$

(Equation 2) $$O_{2}+2H^{+}+2e^{-}\;↔\;H_{2}O_{2}\;E^{0}=0.67\;V\;\text{vs}.\;\text{RHE}$$

(Equation 3) $$O_{2}+4H^{+}+4e^{-}\;↔\;2H_{2}O\;E^{0}=1.23\;V\;\text{vs}.\;\text{RHE}$$

Note, the reversible hydrogen electrode (RHE) is used here which is a pH-dependent scale. The product of the reactions is placed on the right-hand side. Though the 4*e*^*-*^ WOR occurs at a less positive potential, catalysts with a more favorable free-energy reaction pathway toward the 2*e*^−^WOR may still exhibit high selectivity.^3^^,^^11^^,^^12^^,^^13^ However, the high overpotential needed to run 2*e*^−^ WOR limits the initial electrocatalyst selection pool to the materials stable under harsh oxidative conditions. Within this context, previous works have shown SnO_2_, BiVO_4_, WO_3_, boron-doped diamond, and LaAlO_3_ to be effective 2*e*^−^WOR catalysts.^14^^,^^15^^,^^16^^,^^17^^,^^18^^,^^19^ Beyond direct water oxidation, the use of carbonate/bicarbonate species as a mediator to promote 2*e*^−^ WOR has recently been investigated (Figure 1B).^20^^,^^21^ This strategy is promising as its high selectivity can be attained through the selective reaction of oxidized carbonate species with water to yield peroxide, though the exact identity of such reactive intermediates and the mechanism for the overall reaction is not yet established.

Peroxymonocarbonate (HCO_4_^−^; Equations 4 and 5)^13^^,^^21^^,^^22^^,^^23^^,^^24^ and peroxydicarbonate (C_2_O_6_^2−^; Equations 6 and 7)^25^^,^^26^^,^^27^ have been noted as possible intermediates. In addition, radical formation is also a possibility in the highly oxidizing potentials that drive 2*e*^−^ WOR (Equation 12)^28^^,^^29^ and their role as active species has been observed in the presence of transition metals.^30^^,^^31^^,^^32^

(Equation 4) $${\text{HCO}}_{3}^{-}+H_{2}O\;↔\;{\text{HCO}}_{4}^{-}+2H^{+}+2e^{-}\;E^{0}=1.8\;V\;\text{vs}.\;\text{RHE}$$

(Equation 5) $${\text{HCO}}_{4}^{-}+H_{2}O\;\to \;H_{2}O_{2}+{\text{HCO}}_{3}^{-}$$

(Equation 6) $$2{\text{CO}}_{3}^{-}\;\to \;C_{2}O_{6}^{2-}+2e^{-}\;E^{0}=1.8\;V\;\text{vs}.\;\text{RHE}$$

(Equation 7) $$C_{2}O_{6}^{2-}+2H_{2}O\;\to \;H_{2}O_{2}+2{\text{HCO}}_{3}^{-}$$

(Equation 8) $${\text{CO}}_{3}^{\cdot -}+e^{-}\;↔\;{\text{CO}}_{3}^{2-}\;E^{0}=1.57\;V\;\text{vs}.\;\text{RHE}$$

Against this backdrop, we aimed to carry out an investigative work incorporating electrocatalytic and spectroscopic methods to shed light on this process. Using SnO_2_ as a model catalyst, we first probe the role of (bi)carbonate and CO_2_ in mediating 2*e*^−^ WOR. In a complementary direction, we carry out *operando* (i.e., as the reaction is occurring) spectroscopic infrared and Raman experiments that point to the existence of HCO_4_^−^/C_2_O_6_^2−^ species during the reaction process. We observe that a likely limitation of this system is the depletion of near-surface CO_3_^2−^ and subsequently implement pulsed electrocatalysis as a proof-of-concept technique that is used to augment selectivity. In all, the insights derived stand to aid the advent of electrochemical H_2_O_2_ synthesis through the elucidation of key mechanistic aspects underpinning the process.

## Results and discussion

**Figure 2:**
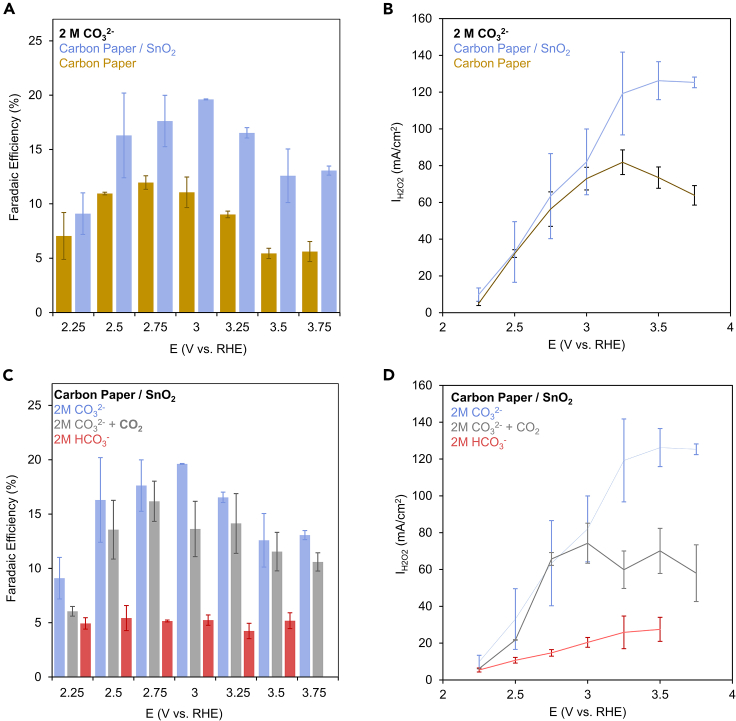
System performance FE (A) and partial current density (B) for H_2_O_2_ production for carbon paper with and without SnO_2_ catalysts in a 2 M CO_3_^2−^ electrolyte point to a beneficial role of the SnO_2_. Further, CO_3_^2−^ is noted as most effective species for mediating the 2*e*^−^ WOR under these conditions as evidenced from the FE (C) and partial current density (D) in comparison with CO_2_ and HCO_3_^−^. Data are represented as mean ± standard deviation.

Our first endeavor entailed establishing a functional 2*e*^−^ WOR system. We used a carbon paper electrode as a reference and SnO_2_ nanoparticles as a catalyst (Figure S2). Using 2 M KCO_3_ as a starting point, we measured the Faradaic efficiency (FE) and partial current density for H_2_O_2_ (I_H2O2_) as a reference point. While this species exists as HO_2_^−^ above pH 11.6, we only use the term H_2_O_2_ for simplicity. In comparing to the bare carbon paper, we noted that the presence of SnO_2_ increased both the FE to a maximum of 20% (Figure 2A) and I_H2O2_ up to 126 mA/cm^2^ (Figure 2B). This indicates that SnO_2_ does not only suppress the 4*e*^−^ WOR but also actively promotes H_2_O_2_ production through facilitating CO_3_^2−^ oxidation.

The potential-dependent FE trends we observe in this work parallel those previously noted for various metal oxides, in which the FE increases with applied potential, then starts to decline.^12^^,^^13^^,^^22^ The current density is also dependent on CO_3_^2−^ concentration, increasing until 0.5 M, and plateauing afterward (Figure S3). The production of H_2_O_2_ was relatively constant with time, decreasing by approx. 5% over the course of 3.3 h (Figure S6) and no obvious changes to catalyst morphology were noted (Figure S7). However, we did see slight changes in the catalyst’s surface electronic structure after such measurements from X-ray photoelectron spectroscopy measurements (Figure S8). A red slight shift (approximately 0.2 eV) in the Sn 3d bands is tentatively attributed to restructuring that may be occurring at the catalyst surface throughout the extended catalytic process.

We then tested the role of CO_2_ and HCO_3_^−^ in mediated H_2_O_2_ production. If the 2 M CO_3_^2−^ electrolyte was saturated with CO_2_ instead of N_2_, the FE and I_H2O2_ both decreased (Figures 2C and 2D). This likely stems from CO_2_ functioning as a spectator species and hindering the diffusion and oxidation of CO_3_^2−^ en route to H_2_O_2_ production. The pH of the solution also drops in this case to a value of approximately 11 so the effects here may be compounded. If 2 M HCO_3_^−^ is used instead, both the FE and I_H2O2_ become significantly lower. This may indicate that HCO_3_^−^ is less effective at mediating the 2*e*^−^ WOR or that it is not active at all. Alternatively, the diminished production that we see stems from CO_3_^2−^ formed in the electrolyte as the pH near the electrode surface drops from H^+^-producing reactions like the 4*e*^−^ WOR. Again, the pH of this solution is lower (approx. 8.6) so pH effects may also play a role (explored in the following section). Interestingly, this is not always the case in the literature as some reports show higher efficiency when HCO_3_^−^ is used instead^3^ and while we do not have a definitive answer to explain these discrepancies, we hope that this work may help close key mechanistic knowledge gaps en route to fully understanding this reaction system and being able to do so.

**Figure 3:**
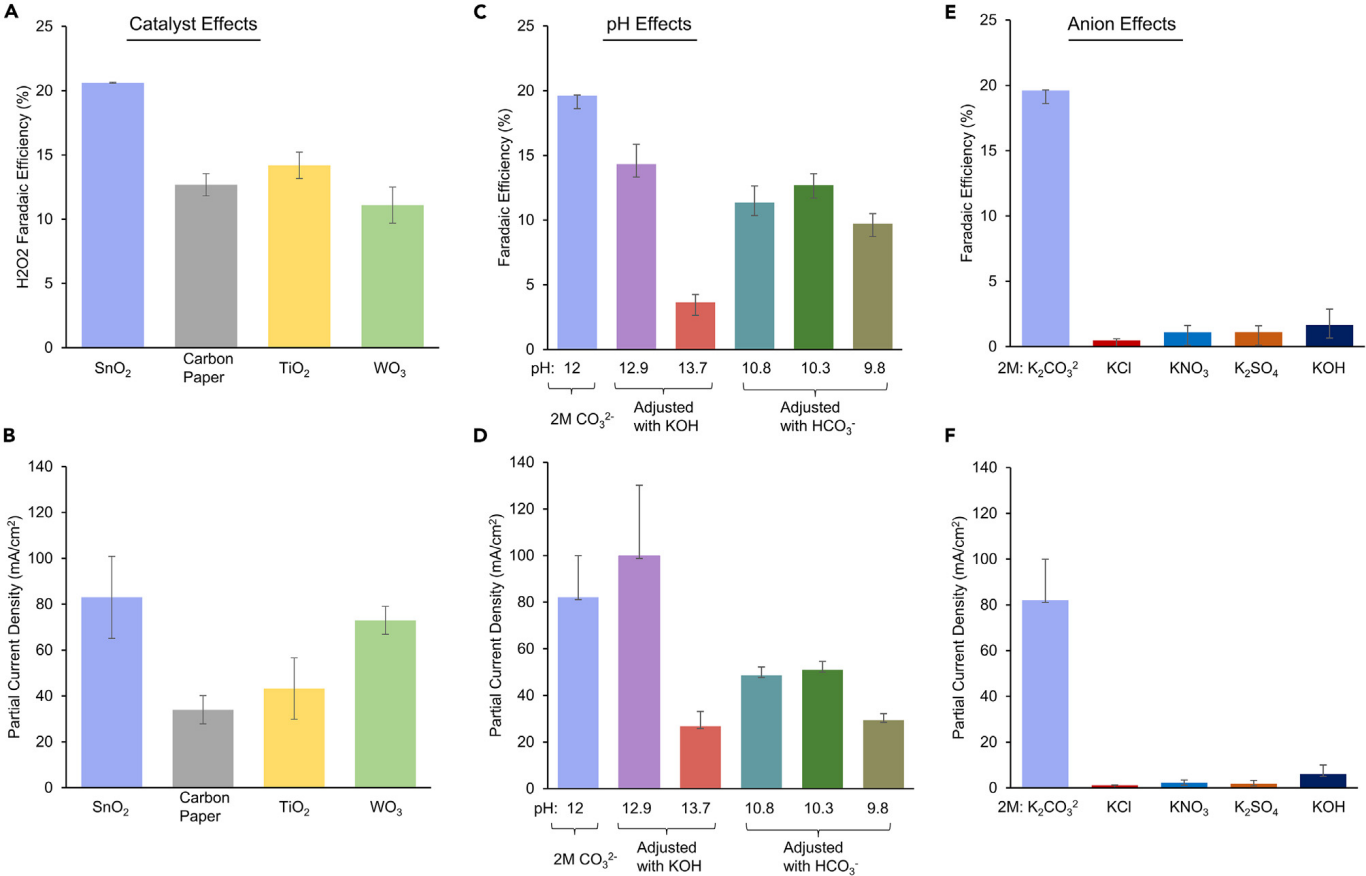
Parameter studies The catalyst placed a role in the system performance as it affected the rate of carbonate oxidation (A, B). The pH was also critical to peroxide production and optimal around 12 (C, D). Finally, the role of other anions as mediators for peroxide production was ruled out as only CO_3_^2−^ ions gave rise to substantial peroxide generation (E and F). Data are represented as mean ± standard deviation.

To deconvolute effects of catalyst, pH, and anions, we performed a series of systematic studies. First, the effects of catalyst surface were probed. We tested the electrochemical system’s performance when SnO_2_ catalysts were replaced either by a bare carbon paper electrode or by equivalent loadings of TiO_2_ or WO_3_ particles under otherwise identical conditions (2M M CO_3_^1−^ at 3 V vs. RHE). We observed that the SnO_2_ electrode substantially outperforms the rest of the electrodes in terms of FE (Figure 3A) and this is tentatively attributed to the CO_3_^2−^ oxidation being more rapid on SnO_2_ surfaces. This notion is also supported by an increase in partial current density with SnO_2_ (Figure 3B). In contrast, WO_3_-loaded electrodes feature lower FE but comparable partial current densities at the same potential and this is attributed to their enhanced activity for the 4*e*^*-*^ WOR.

As we previously observed that CO_2_ and HCO_3_^−^ led to a diminished performance, though the pH was different in the bulk electrolyte, we sought to systematically change the electrolyte pH to measure the resultant system’s performance. At 3 V vs. RHE, we increased the pH with the addition of KOH, while keeping 2 M CO_3_^2−^ in the electrolyte and SnO_2_ as the catalyst (Figures 3C and 3D). We observed that this decreased the FE for peroxide production and this was attributed to an increase in 4*e*^*-*^ WOR activity. Increasing the pH to 12.9 did, however, increase the peroxide partial current density. This signifies that the 2*e*^*-*^ WOR is maximized around pH 12.9 but the selectivity decreases due to competing reactions. Likewise, if the pH was decreased, in this case by adjusting the CO_3_^2−^/HCO_3_^−^ ratio, then the performance decreased once again in terms of FE and partial current density.

We also ruled out the effects of other ions as mediators for the 2*e*^*-*^ WOR. With a SnO_2_ electrode and 2 M concentrations of KCl, KNO_3_, K_2_SO_4_, or KOH as the bulk electrolyte, only minimal activity was observed for peroxide production in terms of FE or partial current density (Figures 3E and 3F). If, for example, we adjusted the pH of the KCl or K_2_SO_4_ electrolyte to 12, the FE remained at 1.1% and 1.6%, respectively, and the partial current densities only reached 1.6 and 2.1 mA/cm^2^. Any peroxide production in this case likely proceeds via a non-mediated pathway.

**Figure 4:**
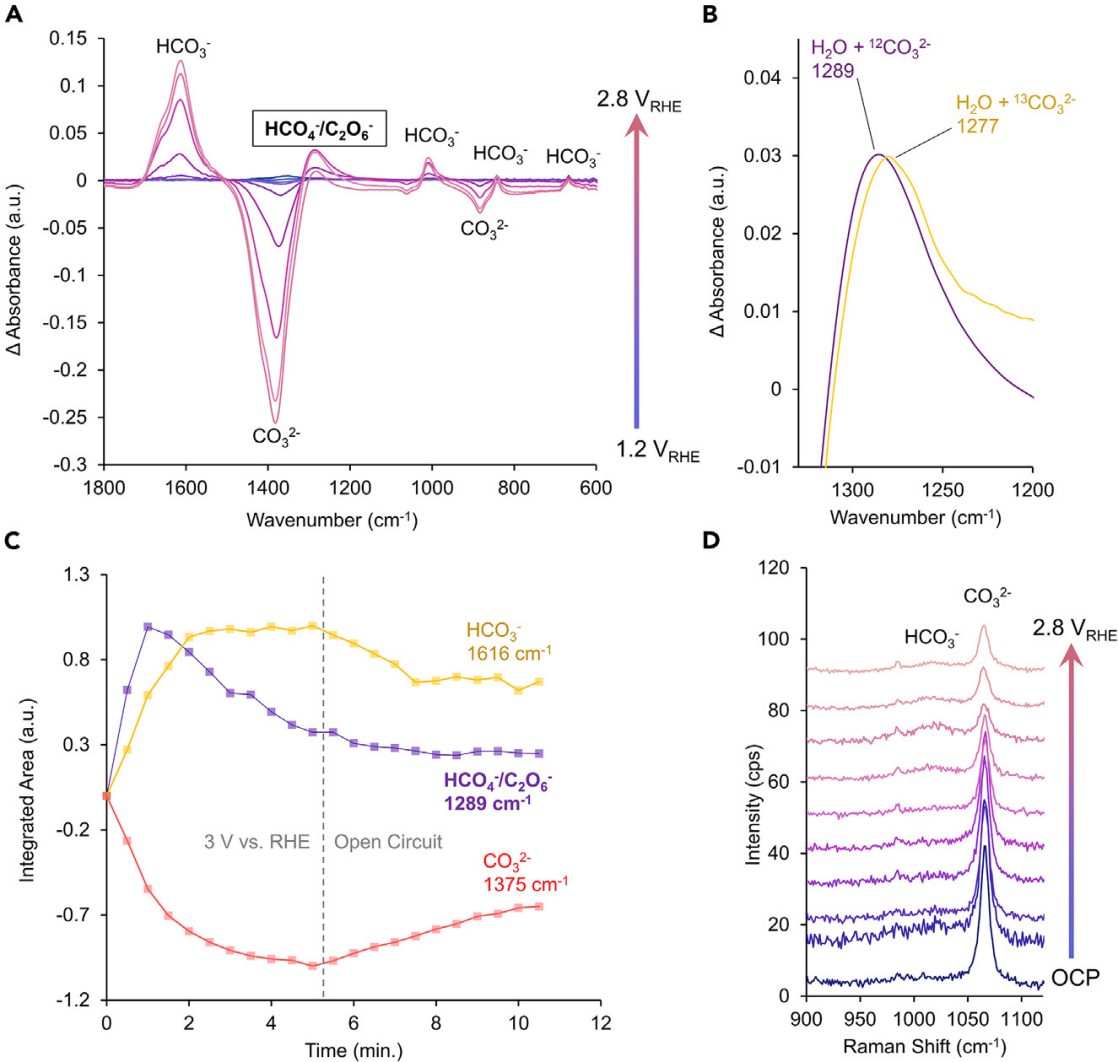
Spectroelectrochemical studies IR spectra measured as a function of applied potential show the depletion of CO_3_^2−^ and formation of HCO_3_^−^ and HCO_4_^−^/C_2_O_6_^2−^ (A), the latter is evidenced through its red-shift under ^13^C-labeled measurements (B). As the potential was stepped from open circuit to 3 V vs. RHE. The rise of HCO_3_^−^ mirrored the consumption of CO_3_^2−^ but the band at 1,288 cm^−1^ attributed to HCO_4_^−^/C_2_O_6_^2−^ reached its maximum much earlier and subsequently decreased (C). SERS spectra show the presence of CO_3_^2−^ and HCO_3_^−^ but surface-bound intermediates were not readily detected (D).

We next turned to vibrational spectroscopy to probe the identity of possible reaction intermediates and active species in the carbonate-mediated 2*e*^−^ WOR. Our initial efforts centered on infrared (IR) spectroscopy, applied under reaction conditions in an external reflection configuration (Figure S9). We noticed that the majority of the spectrum was dominated by the relative changes of the HCO_3_^−^/CO_3_^2−^ species, triggered by under oxidizing potentials that generate H^+^ and lower the pH at the catalyst surface (Figure S10). As such, the HCO_3_^−^ band at 1,616 cm^−1^ grew, indicating the increase of this species while a negative CO_3_^2−^ band 1,375 cm^−1^ also increased in magnitude, indicating the relative diminishment of this molecule (Figure 4A). However, we did see a positive absorption band (indication the formation of a species) at 1,289 cm^−1^. Candidate species in the carbonate-mediated peroxide production mechanism (HCO_4_^−^ and C_2_O_6_^2−^) have previously been noted at this frequency (symmetric C=O stretch), though their exact identity has not been clarified.^33^^,^^34^^,^^35^ To this end, we employed the isotope labeling technique to gain further insights into its origin. Using ^13^C-labeled CO_3_^2−^ led to a 12 cm^−1^ red-shift in this band to 1,277 cm^−1^, indicating that this species likely originated from CO_3_^2−^ oxidation and vibrational mode featured the C-atom from CO_3_^2−^ (Figure 4B). The red-shift was less than that expected for a simple bimolecular C=O stretch and thus this mode may involve multiple atoms. We next probed the temporal evolution of the aforementioned species as the potential was stepped from open circuit to catalytic (3 V vs. RHE). Through acquiring a spectrum every 30 s and integrating the band area, we noted how the negative/positive CO_3_^2−^/HCO_3_^−^ bands growth mirrored each other. This is reasonable as this mainly originates from their direct interconversion. When the potential was turned off (system reverted to open circuit), the magnitude of both bands began to slowly decrease. In contrast to this, the 1,289 cm^−1^ band rose to its maximum at 1 min and decreased afterward, even as the potential remained at 3 V. This led us to believe that this species was rapidly formed, then its quantity decreased, possibly it reacted with H_2_O and additional CO_3_^2−^ was depleted from the direct electrode surface. While the magnitude of HCO_4_^−^/C_2_O_6_^2−^ was much lower than CO_3_^2−^, this measurement probed not only the surface but also within 1–2 μm away, rendering it difficult to directly correlate the bands to what is present directly at the electrode surface.

To this end, a subsequent question arose in determining whether this species was a surface-bound intermediate or a solution-based mediator. To address this challenge, we turned to surface-enhanced Raman spectroscopy (SERS) (Figure S13). Because of the surface-sensitive nature of the SERS technique, we hypothesized that surface-bound intermediates would be detected with SERS and IR measurements while near-surface species would only be detected with IR experiments.

We fabricated SERS-active electrodes through a simple electrodeposition and galvanic exchange approach.^36^ The resulting catalysts consisted of an Au underlayer with a SnO_x_ coating that mimics the SnO_2_ catalysts used as previously described (Figure S14). As SERS selectively provides information on chemical species within a few nm of the surface, this would reveal if any substantial quantities of surface-bound species would be built up under reaction conditions. However, upon the application of successively oxidizing potentials, the main species detected were CO_3_^2−^, and HCO_3_^−^, present near the surface (Figures 4D and S14). CO_3_^2−^ decreased as it was depleted via its oxidation and from the interconversion to HCO_3_^−^ as the pH decreased. We did not see evidence of surface-bound species of HCO_4_^−^/C_2_O_6_^2−^, which would have expected to yield strong bands around 900 and 700 cm^−1^ even as we continually observed (bi)carbonate species in the spectra.^34^^,^^35^^,^^37^^,^^38^ We took this as evidence for carbonate-derived species rapidly diffusing away from the surface and reacting in the bulk of the solution to yield hydrogen peroxide following their oxidation. This is consistent with reports that show the continual formation of peroxide for several minutes following the end of electrolysis, as well as rotating ring disk electrochemical measurements that show a peroxide-oxidation current that is rotation speed (and consequently mass transport) dependent.^39^ If there was any surface-bound species beyond bi(carbonate), it was likely very short-lived and rapidly desorbed and thus its population is too low to be detected.

**Figure 5:**
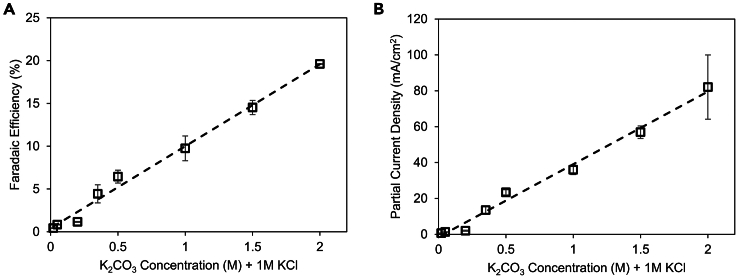
Concentration dependence The FE (A) and partial current density (B) for peroxide production both followed a linear relation with the concentration of CO_3_^2−^ in the electrolyte, indicating first-order kinetics. Data are represented as mean ± standard deviation.

Because HCO_4_^−^ and C_2_O_6_^2−^ both feature a distinct absorption band around 1,289 cm^−1^ that is noted in our spectra, we sought an alternative route to provide evidence for the identity of the primary CO_3_^2−^-derived species. Because HCO_4_^−^ requires one CO_3_^2−^ reactant while C_2_O_6_^2−^ needs CO_3_^2−^ species to form, we hypothesized that the peroxide production rate would follow either a linear relationship in the case of a HCO_4_^−^-mediated mechanism or exponential relationship if the C_2_O_6_^2−^-mediated mechanism prevailed. The other reactant, water, is assumed to be in excess. To this end, we performed a series of systematic experiments that measured both the FE and partial current density for peroxide as a function of CO_3_^2−^ (Figure 5). Previously identified optimal conditions (2 V vs. RHE) were used, with SnO_2_ catalysts. 1 M KCl was used as a supporting electrolyte so electrolyte conductivity did not significantly vary across experiments. Across a wide concentration range, we observed a clear linear relationship in both FE and reaction rate and thus, we tentatively postulate that HCO_4_^−^ is the key intermediate in our system. While this is not unambiguous proof, this assignment is consistent with our dataset.

**Figure 6:**
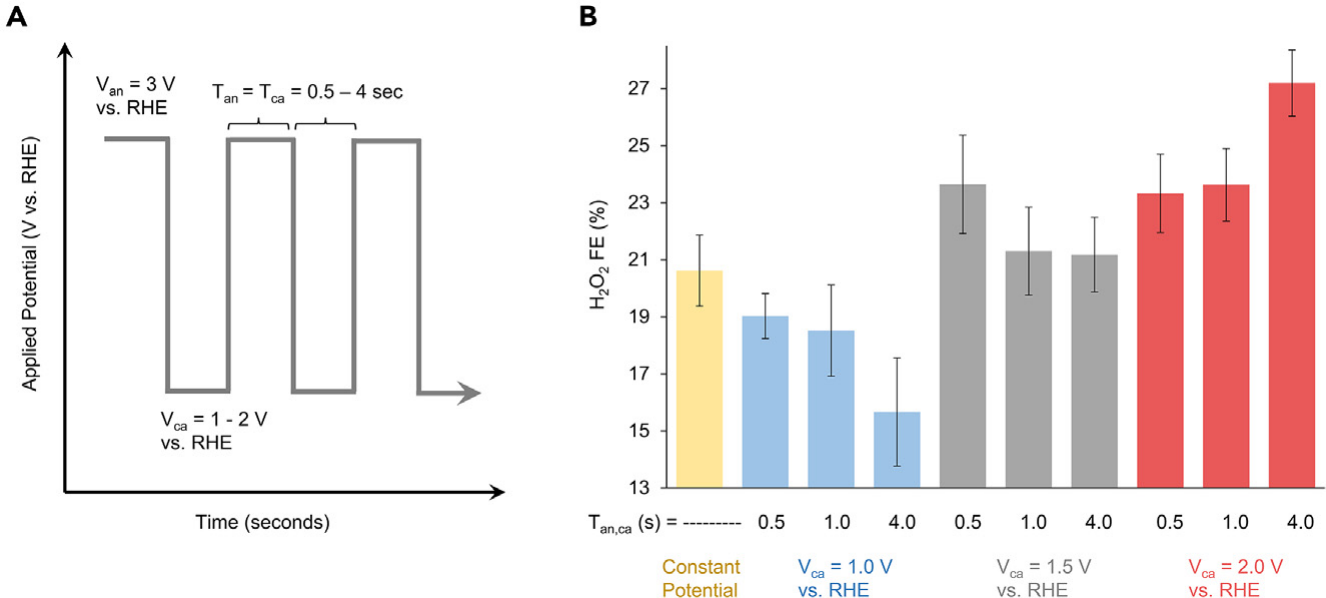
Pulsed electrolysis Schematic of pulsed-potential electrolysis (A) and summary of peroxide FE under different V_ca_ and pulse durations (B). Data are represented as mean ± standard deviation.

From our experiments detailed previously, we believe that the depletion of CO_3_^2−^ from the catalyst near-surface region is one of the limitations hindering the selectivity of the system. To see if we could, in part, circumvent this, we turned to the application of pulsed electrocatalysis. This technique entails the alternate applications of two or more different potentials as a function of time (Figure 6A), contrasting the constant-potential electrolysis that we have been using up to now. This technique has been successfully implemented in organic synthesis^40^ and heterogeneous electrocatalysis,^41^ though not yet in the context of CO_3_^2−^-mediated peroxide synthesis. While this method can modulate reaction mechanisms by instituting a secondary reaction step^42^ or alter the catalyst structure,^43^ we sought to simply use the anodic voltage, V_an_, to carry out catalysis and the cathodic voltage, V_ca_ (simply named for convenience as little current was passed), to enable the carbonate species to diffuse to the electrode surface and replenish the reactants available. In particular, we hypothesized that during non-reactive portions of the experiment, the replenishment of the near-surface region with reactants (CO_3_^2−^) would yield higher selectivity.

We also investigated several pulse durations (t_an_, t_ca_) from 0.5 to 4.0 s, keeping the anodic and cathodic times equivalent. Modulating the pulse durations would determine the time allowed for near-surface concentrations of reactants and ions to equilibrate. The previously optimized V_an_ was used as that is where the FE was the highest in constant potential measurements. V_ca_, in contrast, was modulated in an effort to minimize back-reactions while still allowing for re-equilibration of CO_3_^2−^ concentrations.

We first saw that using a V_ca_ of 1.5 V was detrimental to the system, no matter what pulse times were used (Figure 6B). We believe that the root cause of this was that at this potential, reactive species of HCO_4_^−^/C_2_O_6_^2−^ or their derivatives may be reduced back to CO_3_^2−^. When V_ca_ was increased to 1.5 V vs. RHE, we saw an increase of FE. In this regime, there may still be some re-oxidation of intermediates but the increase stems from a more efficient replenishment of CO_3_^2−^ to the electrode surface. Having a shorter pulse duration was more beneficial in this case as perhaps this minimized back-reduction of products while still enabling adequate diffusion. Finally, when V_ca_ was set to 2.0 V vs. RHE, we obtained the best results, yielding an FE increase up to 27%. We believe that the re-reduction is likely minimized here. In addition, having a longer pulse duration of up to 4s for both the cathodic and anodic pulses that yield the highest peroxide selectivity may enable more reactants to diffuse to the surface.

There was minimal current passed during the pauses in catalysis during the pulsing experiments and the anodic, catalytic current for pulsed and non-pulsed experiments did not vary significantly (Figure S17). Thus, we believe that changes in the species at the near-surface region shifted the catalysis from water oxidation to O_2_ to the carbonate-mediated peroxide synthesis. There may be opportunities to push the selectivity for peroxide even higher through the use of electrolytes/solvents/conditions with even higher CO_3_^2−^ solubility.

### Limitations of the study

While we did not explore asymmetric pulses, sinusoidal-shaped potential vs. time applications, or multi-potential pulses as this is outside of the scope of the current work, these methods may yield even more substantial FE increases. In addition, we do not have direct proof that the difference in performance through pulsed electrolysis results from balancing mass transport and minimizing re-reduction of intermediates and this would be an interesting question to explore with time-resolved methods beyond our scope. The same goes for the infrared experiments, in which a new band not attributable to (bi)carbonate was detected though not unambiguously assigned, although we postulate it to be that from HCO_4_^−^ from the first-order dependence of partial current density on CO_3_^2−^ concentration. While the short-lived nature of this species renders it difficult to isolate and fully characterize, additional complementary techniques like electron paramagnetic resonance may prove beneficial in constructing a more comprehensive mechanistic picture. Experimental data such as performance across different catalysts/conditions and spectra from spectroelectrochemical experiments would further be strengthened through matching with predictions derived from computational modeling. We also demonstrated differences in activity with different catalysts and an open question remains as to why this is the case. While CO_3_^2−^ oxidation is thought to be an outer-sphere reaction, differences in surface roughness, adsorption of chemical species (e.g., OER intermediates), double-layer composition, and more can potentially all influence the rate of the reaction and this is yet to be explored.

For the translation of practical systems, these findings imply that effective electrolyte flow near-surface mass transport is key toward maximizing selectivity toward peroxide production. Beyond this, there are certainly additional challenges that need to be solved like product separation, scale-up, and full system integration and we encourage researchers to consider how individual findings fit within progressing the field as a whole.

### Concluding remarks

In this work, we investigated the carbonate-mediated oxidative electrosynthesis of peroxide from water with a combination of electrochemical and spectroscopic techniques. In our system, the results indicate the CO_3_^2−^, and not HCO_3_^−^ or CO_2_. is the dominant reactant leading to H_2_O_2_ production. Following up with spectroscopic measurements, we provide evidence for the existence of a CO_3_^2−^-derived species, likely HCO_4_^−^/C_2_O_6_^2−^, whose production may be limited by the depletion of near-surface CO_3_^2−^. Finally, we help to circumvent this issue through the application of pulsed electrolysis that enables the CO_3_^2−^ reactants to diffusion back to the electrode surface. The insights from this work stand to help guide the development of practical H_2_O_2_ electrosynthesis technologies.

## STAR★Methods

### Key resources table

**Table:** 

REAGENT or RESOURCE	SOURCE	IDENTIFIER
**Other**		

Toray carbon paper	Thermo Scientific	CAS 7782-42-5
Tin (IV) oxide nanopowder	Sigma-Aldrich	100 nm, CAS 18282-10-5
TiO_2_	Sigma Aldrich	(<100 nm particle size, CAS 13463-67-7
Tungsten(VI) oxide	Sigma Aldrich	(<100 nm particle size, CAS1314-35-8

### Resource availability

#### Lead contact

Further requests for resources and information can be sent to nkornien@uni-bonn.de.

#### Materials availability

This study did not generate new unique reagents.

#### Data and code availability

**Data:** Data reported in this paper will be shared by the lead contact upon request.

**Code:** This study did not generate any original code.

**Additional:** Any additional information required to reanalyze the data reported in this paper is available from the lead contact upon request.

### Method details

Electrochemical measurements were performed at room temperature and atmospheric pressure by BioLogic SP-200 potentiostat/galvanostat. An Ag/AgCl/KCl_sat_ was used as the reference electrode, and graphite as the counter electrode, separated from the working and reference electrodes by a glass frit in a separate chamber. All of the scan rates are fixed at 10 mV sec^-1^.

H_2_O_2_ production was calculated based on previously established colorimetric methods. A light yellow solution of 5 mM of cerium(IV) sulfate and 1 M H_2_SO_4_ was prepared, which in the presence of peroxides turns colorless. 1 mL of peroxide solution with known concentrations were added to 3 mL of the yellow cerium solution, then the color changes were quantified by UV-Vis absorption spectroscopy.

A catalyst ink consisted of 10 mg SnO_2_, 100 μL water, 300 μL ethanol, and 25μL of Nafion (5% wt. in water/propanol) was thoroughly mixed by sonication. To prepare the working electrode, each side of the carbon paper substrate was coated with the prepared ink to arrive at a total SnO_2_ loading of 1 mg/cm^2^. For electrochemical and Raman measurements, SnO_2_ coated on carbon paper was used.

Infrared spectroscopy was performed using a Nicolet 380 by Thermo Scientific. Spectroelectrochemical measurements were performed in external reflection mode, with a carbon counter and Ag/AgCl reference electrode. SnO_2_ electodes were prepared by drop casting SnO_2_ onto flexible carbon cloth electrodes that were more suitable for this geometry. We note that using the external reflection geometry may lead to potential deviations from catalytic conditions used because mass transport to the catalyst surface may be limited and that the cell resistance and thus the IR drop between the working and reference electrode may be larger. This should be taken into account when interpreting spectroscopic data.

Surface enhanced Raman spectroscopy was performed on Renishaw inVia Raman spectrometer with a 633 nm laser source. For these measurements, Au dendrites were first grown on carbon paper electrodes through electrodeposition at -0.4V vs. Ag/AgCl (3 minutes) in a 0.5 M HCl and 0.2 M AuCl_3_ electrolyte. After rinsing with water, a sacrificial Zn layer was grown using 0.1 M phosphate buffer (pH 7) with 1 mM ZnNO_3_ (2 minutes at -1.2 vs. Ag/AgCl). After rinsing with water, the electrode was soaked in a saturated solution of SnCl_2_ for 30 minutes to carry out a galvanic exchange process.

## Supplemental information

Document S1. Figures S1‒S17
