# Supplementary material for: Combined electrochemical and spectroscopic investigations of carbonate-mediated water oxidation to peroxide
Source: iScience. 2024 Mar 11;27(4):109482. doi: 10.1016/j.isci.2024.109482 (PMC10981096; doi:10.1016/j.isci.2024.109482)
Supplement: Document S1. Figures S1‒S17 [file mmc1.pdf]

iScience, Volume *27*

Supplemental information

Combined electrochemical and spectroscopic investigations of carbonate-mediated water oxidation to peroxide

Hossein Bemana and Nikolay Kornienko

Supporting Information

Combined Electrochemical and Spectroscopic Investigations of Carbonate-Mediated Water Oxidation to Peroxide

Hossein Bemana[1,2] and Nikolay Kornienko[1,2]*

[1]Department of Chemistry, Université de Montréal, 1375 Avenue Thérèse-Lavoie-Roux, Montréal, QC H2V 0B3, Canada.

[2] Institute of Inorganic Chemistry, University of Bonn, Gerhard-Domagk-Str. 1, 53121 Bonn, Germany

*email at nkornien@uni-bonn.de

Supplemental Experimental Procedures

Methods and materials

The commercially available materials were used without further purification: Toray carbon paper from Thermo Scientific (CAS 7782-42-5), CeTech Carbon Cloth with MPL from Fuelcell Store (W1S1011), Tin (IV) oxide nanopowder from Sigma-Aldrich (<100 nm, CAS 18282-10-5), TiO_2 from Sigma Aldrich (<100 nm particle size, CAS 13463-67-7), Tungsten(VI) oxide from Sigma Aldrich (<100 nm particle size, CAS1314-35-8), K_2CO_3 from VWR Chemicals (CAS 584-08-7), $KHCO_3$ from Alfa Aesar (CAS 298-14-6), K_2CO_3-^{13}C from Sigma Aldrich (CAS 122570-45-0), $NaHCO_3$-^{13}C from Sigma Aldrich (CAS 87081-58-1), Deuterium Oxide D_2O from Sigma Aldrich (CAS 7789-20-0).

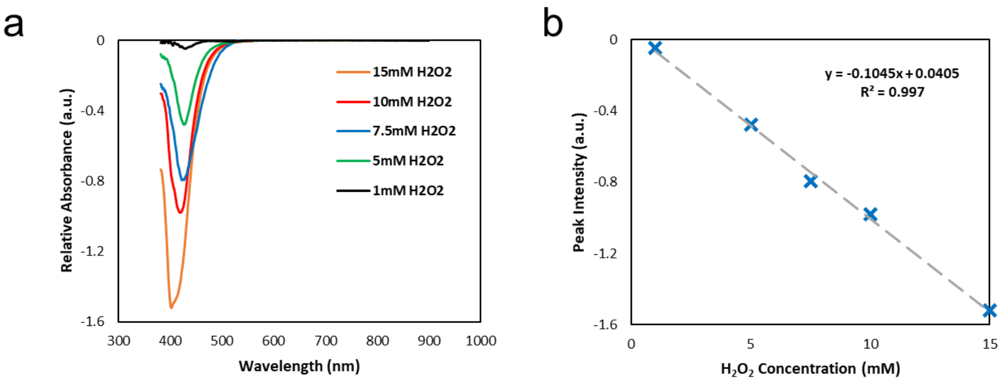

Figure S1. Colorimetric quantification calibration. UV-Vis spectra illustrating the change in absorbance (decrease around 400 nm) with increasing H_2O_2 concentrations (a) and calibration curve (b).

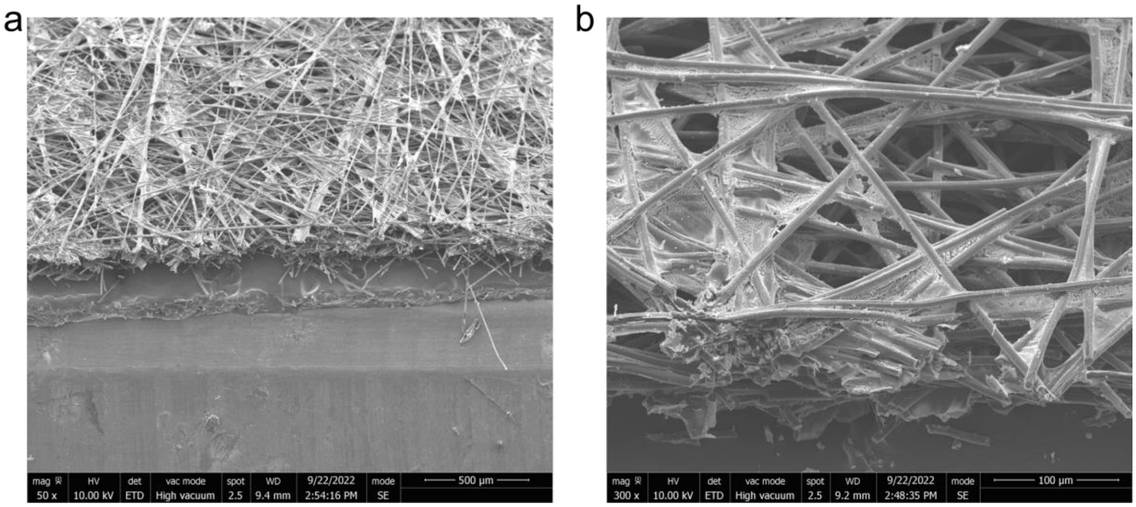

Figure S2. SEM analysis of electrodes. Carbon paper electrodes coated with SnO_2 catalysts under low (a) and high (b) magnification.

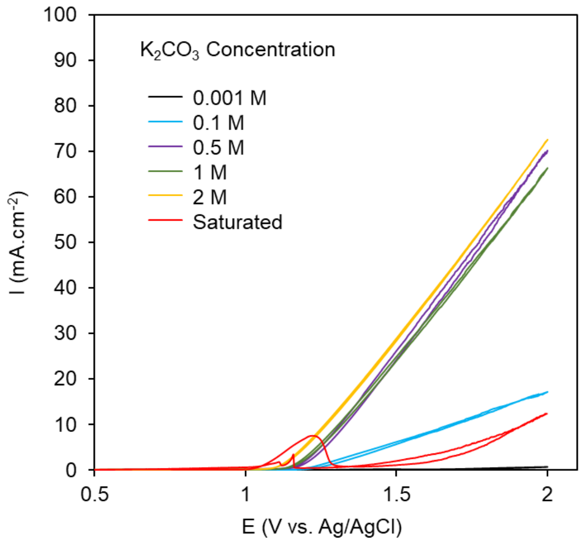

Figure S3. Electrochemical testing of carbonate oxidation. CVs of the carbon paper/SnO_2 electrode with increasing concentration of CO_3^{2-} in the electrolyte.

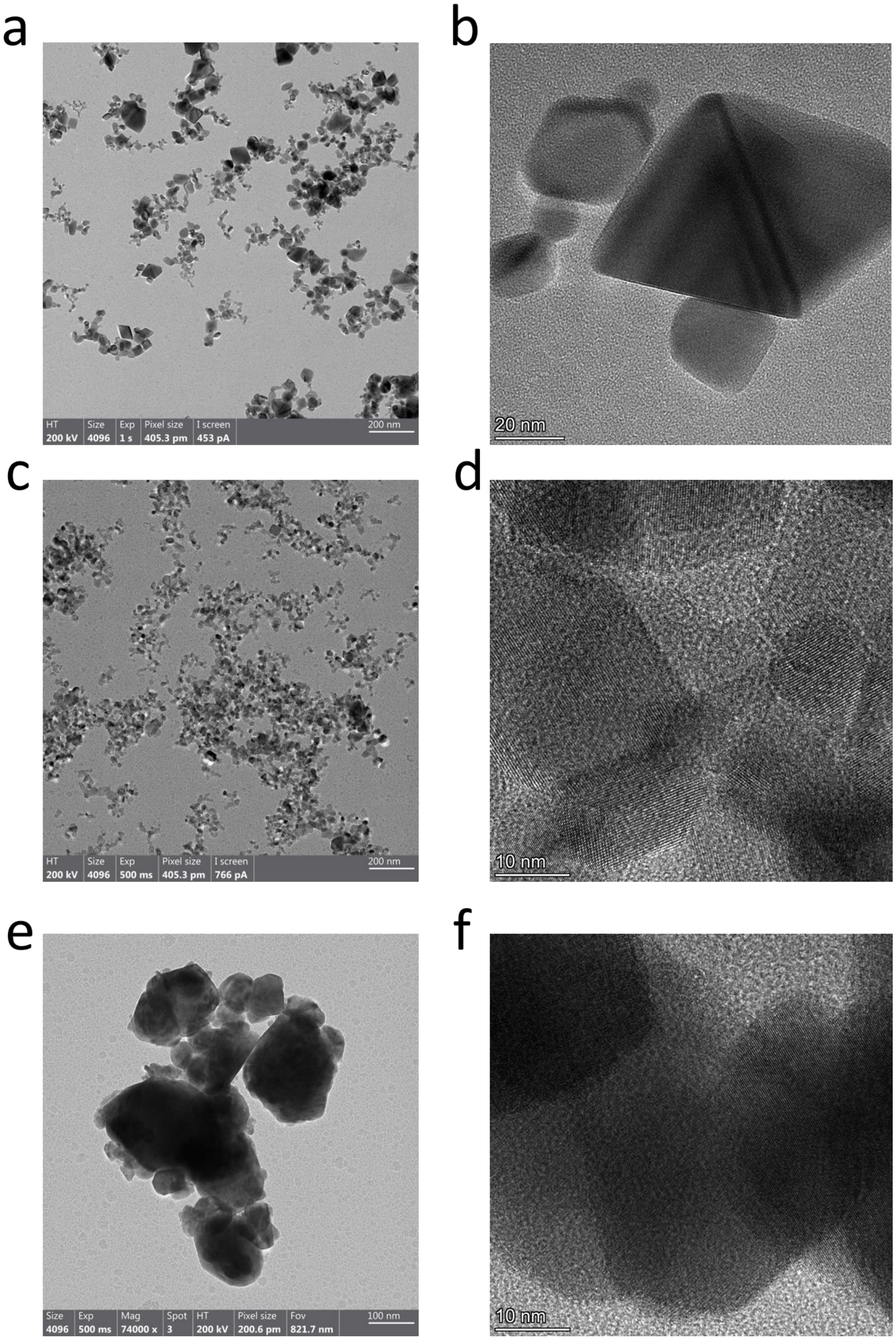

Figure S4. TEM analysis of catalyst particles. TEM images of SnO_2 (a, b), TiO_2 (c, d), and WO_3 (e, f) catalysts used in this work.

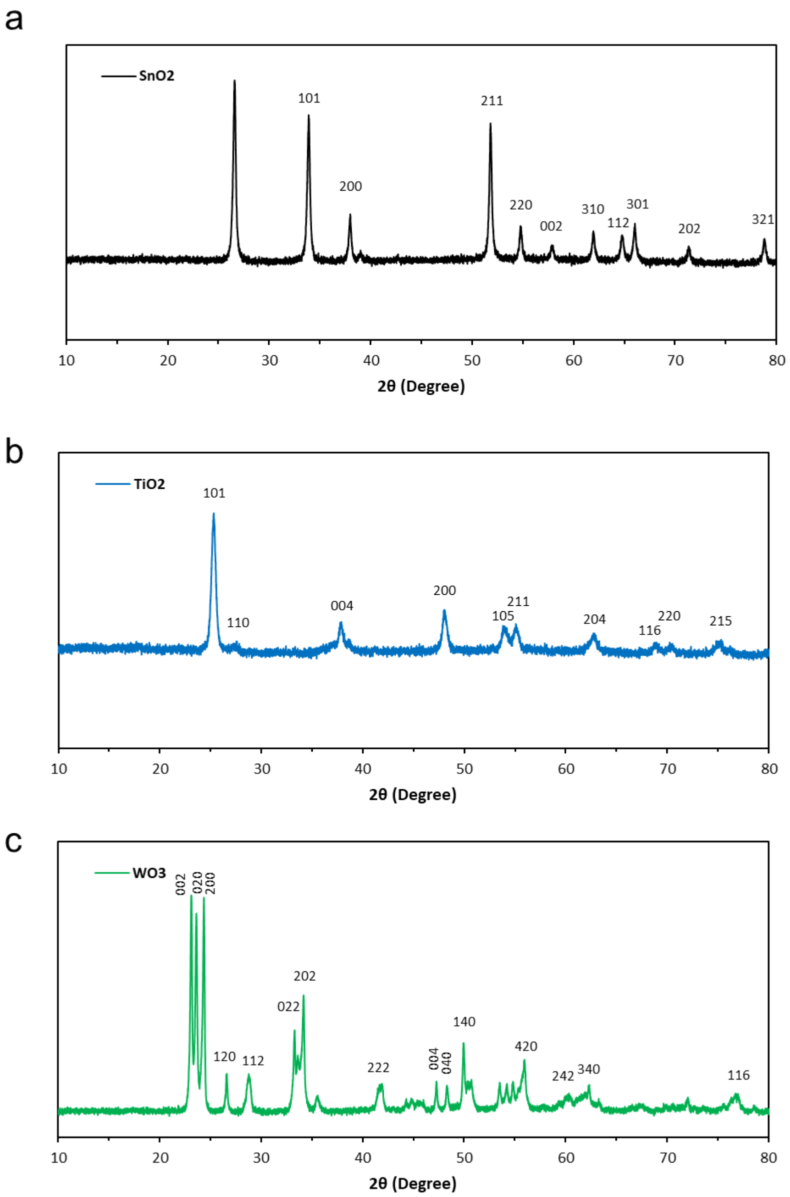

Figure S5. Crystal structure analysis of catalysts. XRD patterns of SnO_2 (a), TiO_2 (b), and WO_3 (c) catalysts used in this work.

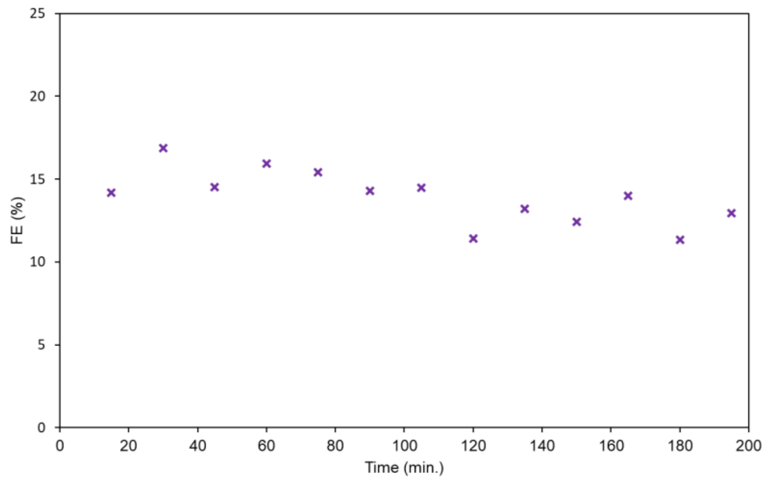

Figure S6. Stability measurements. FE for H_2O_2 production over time with SnO_2 at 3V vs. RHE

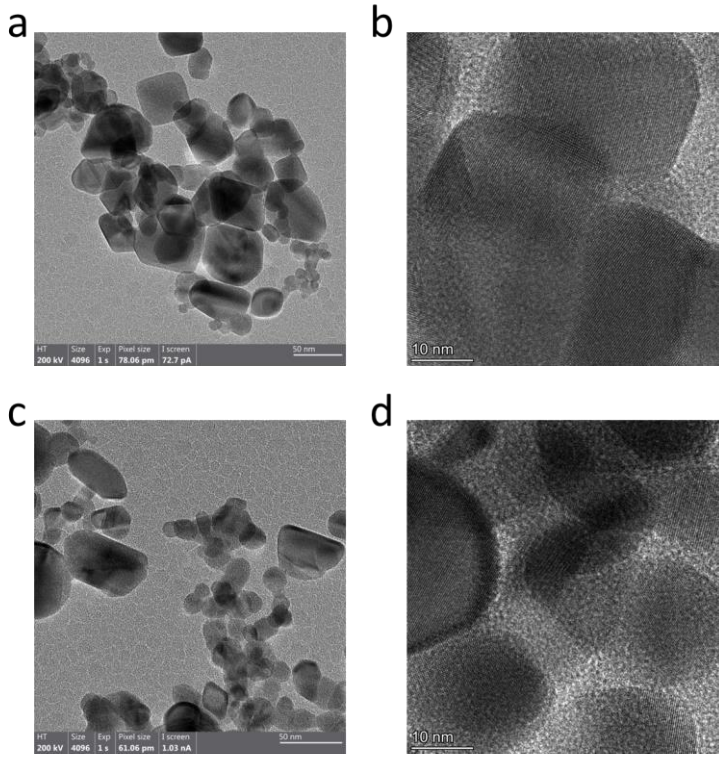

Figure S7. Investigation of morphological catalyst changes. TEM images of SnO_2 particles prior to their use (a, b) and after 1 hr of catalysis at 3 V vs. RHE (c, d).

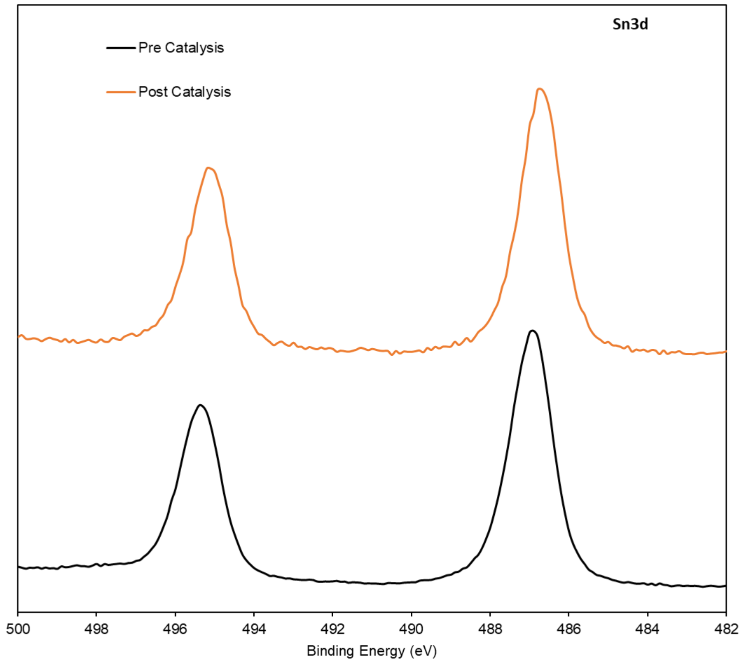

Figure S8. XPS analysis of surface changes. XPS spectra of SnO_2 electrodes prior to their and after 1 hr of catalysis at 3 V vs. RHE. A slight shift by approximately 0.2 eV is noted and tentatively attributed to surface restructuring throughout the catalytic process.

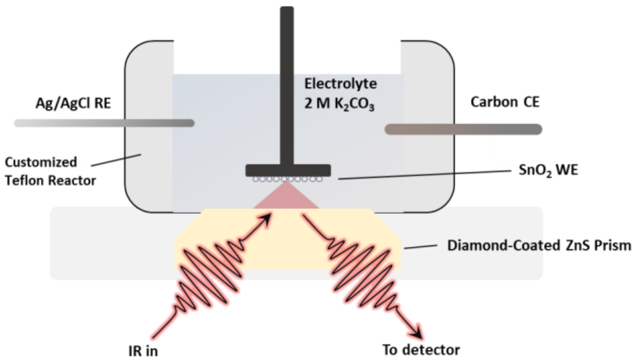

Figure S9. Spectroelectrochemical setup. Simplified diagram of spectroelectrochemical cell used for IR measurements

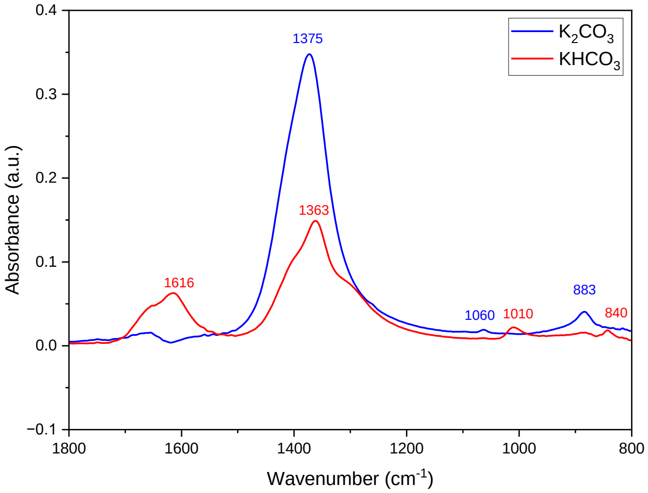

Figure S10. Standard solution spectra. IR spectra of K_2CO_3 and $KHCO_3$ dissolved in H_2O (using H_2O as a background).

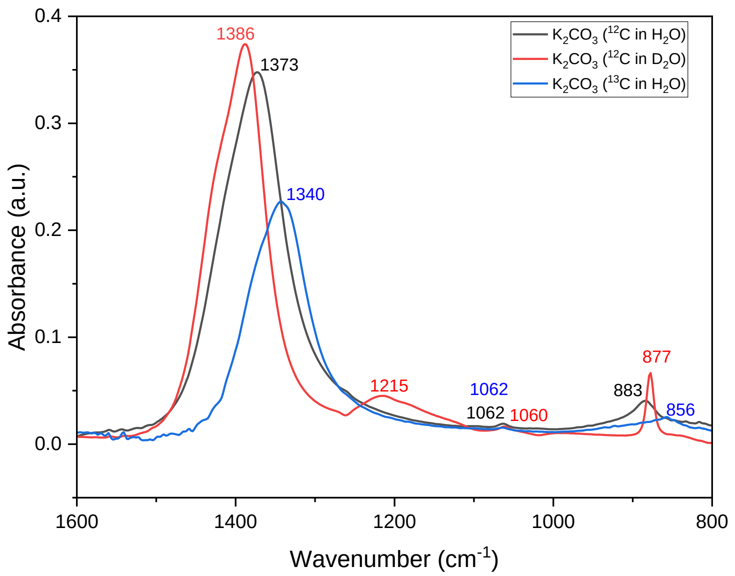

Figure S11. Isotopic shifts. IR spectra of K_2CO_3 in H_2O, D_2O and ^{13}C-labelled K_2CO_3 in H_2O

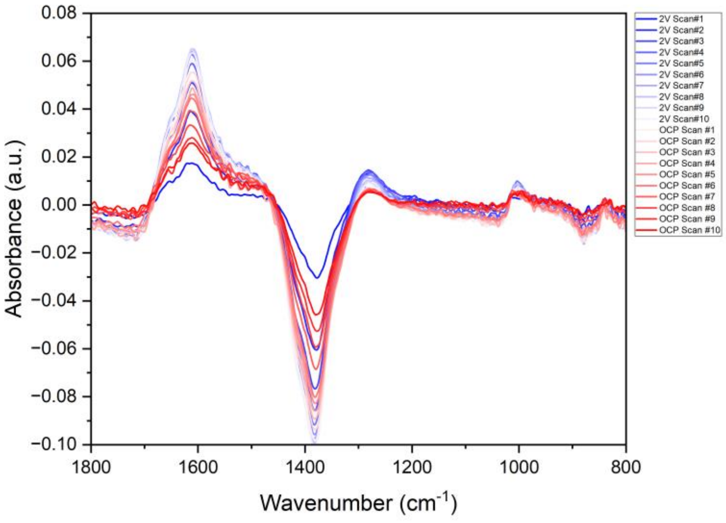

Figure S12. Time dependent spectroelectrochemistry. IR spectra taken as a function of time. Each scan lasted 30 seconds (10 minutes total run time), going from 2V vs. Ag/AgCl for 5 minutes to open circuit for another 5 minutes, with an initial spectrum at open circuit serving as the backgdound.

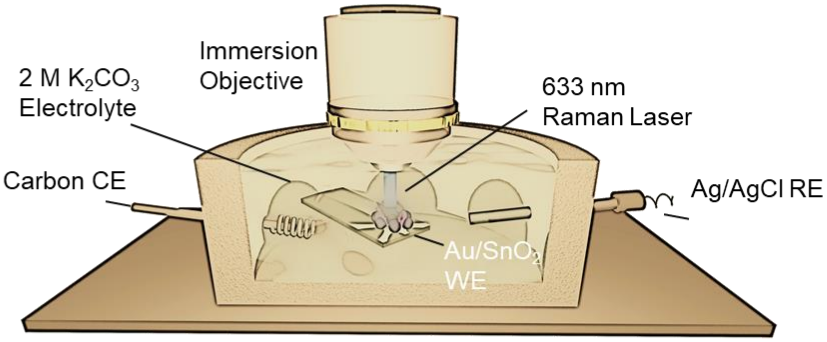

Figure S13. Raman cell configuration. Spectroelectrochemical Raman setup used in this work

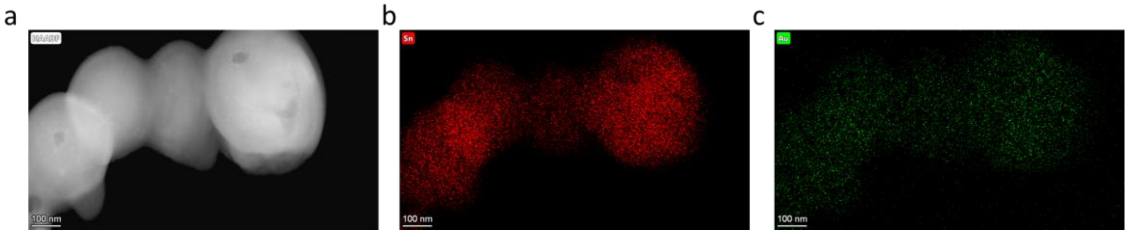

Figure S14. **Materials used for Raman measurements.** STEM image of the Au/Sn structures used for Raman spectroelectrochemical measurements (a), and elemental mapping of Sn (b) and Au (c) of the structures.

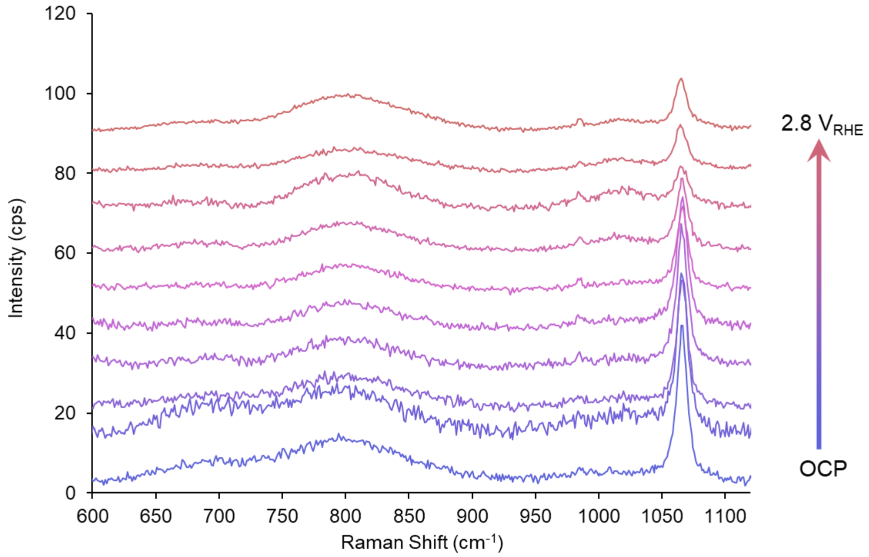

Figure S15. **Raman spectra under applied voltages.** Potential-dependent Raman spectra of CO_3^{2-} oxidation (2M K_2CO_3 electrolyte),

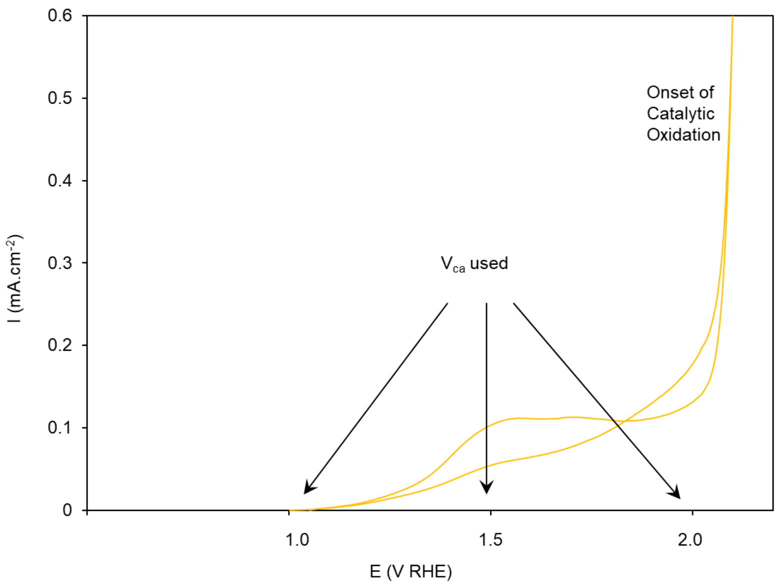

Figure S16. **CV illustration.** CV of the carbon paper/SnO_2 catalyst in 2 M K_2CO_3 electrolyte, with the potential (V_{ca}) used in pulsed electrosynthesis experiments.

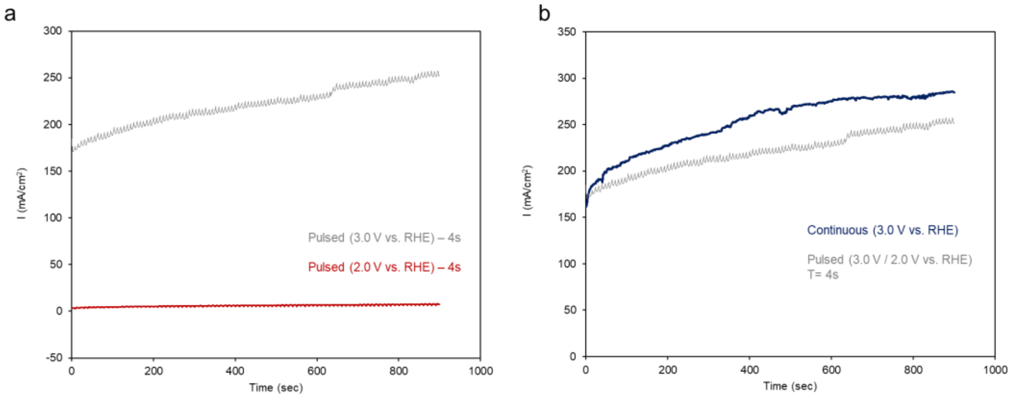

Figure S17. **Current vs. time for puled and static measurements.** Current vs. time plot of typical pulsed electrolysis experiment (a) and a comparison of cathodic current of pulsed and continuous electrolysis at 3.0 V vs. RHE (b).
